# Innate-immune cell distribution in pediatric HIV patients and uninfected controls

**DOI:** 10.1590/S1678-9946202466075

**Published:** 2024-12-16

**Authors:** Cynthia Oliveira Aquino, Fernanda Mariz Pereira, Ana Cristina Cisne Frota, Cristina Barroso Hofer, Lucimar Gonçalves Milagres, Wânia Ferraz Pereira Manfro

**Affiliations:** 1 Universidade do Estado do Rio de Janeiro, Departamento de Microbiologia, Imunologia e Parasitologia, Rio de Janeiro, Rio de Janeiro, Brazil; 2 Universidade Federal do Rio de Janeiro, Instituto de Puericultura e Pediatria Martagão Gesteira, Rio de Janeiro, Rio de Janeiro, Brazil; 3 Universidade Federal do Rio de Janeiro, Departamento de Medicina Preventiva, Rio de Janeiro, Rio de Janeiro, Brazil

**Keywords:** HIV, Children; monocytes, Natural killer cells, Myeloid-derived suppressor cells

## Abstract

Innate immune cells are important players during an infection. The frequency of monocytes, myeloid-derived suppressor cells (MDSCs), natural killer (NK), and NKT cells were assessed in blood samples of children and adolescents living with HIV (CALHIV) and HIV-uninfected (HU) children. Blood samples from 10 CALHIV (treated or not) and six HU individuals were collected for approximately one year. Flow cytometry was employed to phenotypically characterize cell populations. We found a lower frequency of classical monocytes in CALHIV patients compared to the HU group (35.75% vs. 62.60%, respectively) but a higher frequency of CD56^−^CD16^+^ NK cells in CALHIV patients compared to the HU group (1.45% vs. 0.44%, respectively). At baseline, the frequency of monocytic-MDSCs inversely correlated with CD56^dim^CD16^+^ NK cells (r= −0.73, p=0.020), CD56^−^CD16^+^ NK cells (r= −0.78, p=0.010), and with intermediate monocytes (r= −0.71, p=0.027) in the CALHIV group. We also found a negative correlation between CD56^++^CD16^+−^ and CD56^dim^CD16^+^ NK cells with CD4 T cells frequency at baseline. The results suggest an alteration in the innate compartment of the CALHIV cohort, which may contribute to their susceptibility to infections.

## INTRODUCTION

Human immunodeficiency virus (HIV) infection disrupts both the innate and adaptive immune system, increasing children's susceptibility to infections and impairing their ability to respond effectively to vaccines^1^. Although combined antiretroviral therapy (cART) effectively suppresses viral replication, inflammation persists at elevated levels in treated patients, thus compromising their immune response^2^.

The innate immune system plays a crucial role in recognizing antigens and shaping the nature of the adaptive immune response^3^. Monocytes can be activated by various pathogen molecules and secrete a wide range of cytokines. During inflammation, they migrate to tissues and undergo differentiation into macrophages^4^. Natural killer (NK) cells contribute to the antiviral response via cytokine production or direct killing of infected cells^5^. An HIV-infected cell becomes NK-target by upregulation of surface molecules or by inhibiting the expression of major histocompatibility complex (MHC) class I^6^. Additionally, natural killer T (NKT) cells play a role in controlling viral infections by producing inflammatory cytokines and cytotoxicity while also promoting immune regulation^7^.

On the other hand, myeloid-derived suppressor cells (MDSCs) represent a heterogeneous population expanded during HIV infection. These cells can suppress immune response via different mechanisms, favoring HIV replication^8^. Notably, a crosstalk among these innate immune cells occur, as a crosstalk with the adaptive immune system also occurs.

Children living with HIV are more susceptible to infections caused by encapsulated bacteria such as *Neisseria meningitidis* ^9,10^ and present a weaker response to vaccines compared to uninfected children^11–13^.

The studies on immune response during HIV infection are mainly focused on the adaptive immune system. However, it is crucial to recognize that innate immune cells represent the frontline defense against infections. We evaluated the kinetics of select innate immune cells in children and adolescents living with HIV (CALHIV), both under cART or not, and noninfected children and adolescents over a follow-up period of approximately one year. We took advantage of a previous cohort of HIV+ children and adolescents immunized with two doses of meningococcal C conjugate vaccine (MCC) to perform these evaluations, which enabled the longitudinal monitoring of cell frequencies^13^.

## MATERIALS AND METHODS

### Study population and sample collection

This study is part of an original investigation conducted at Instituto de Puericultura e Pediatria Martagao Gesteira, Universidade Federal do Rio de Janeiro (IPPMG/UFRJ). Eligibility criteria for the original study included: age from 2 to 18 years old; no previous MCC immunization; no symptoms or diagnosis of other immunosuppressive disease; no use of systemic immunosuppressive medications; and no history of hypersensitivity to MCC components. For the CALHIV group, additional criteria included a CD4 T cell count ≥350 cells/mm^3^ and/or 15% at the beginning of the study. For HIV-uninfected individuals, a negative HIV serology after 18 months of age was required. Details about inclusion and exclusion criteria, as well as ethical approval, have been previously published^14^.

In total, 10 CALHIV (6 on cART) and six HIV-uninfected (HU) individuals were enrolled in this study. Among the CALHIV and HU groups, 60% and 33.3% participants were female, respectively. HU group comprises individuals assisted at IPPMG. The samples were chosen according to the availability of stored peripheral blood mononuclear cells (PBMCs). HU and CALHIV groups received one or two doses of the MCC vaccine (Novartis; C polysaccharide/CRM_197_), respectively. Blood samples were collected before immunization (T0, baseline) and 1–2 months after the first dose (T1) for both groups. The CALHIV group received a second dose (T2) about one year after the first MCC dose, with blood samples collected both at T2 and 1–2 months following the second dose (T3) (Supplementary Figure S1A). PBMCs were obtained as described elsewhere^15^. Clinical analyses, such as CD4 count and viral load, were conducted at IPPMG during each sample collection (T0–T3). Clinical data were retrieved from patient medical records^13^. All the samples were collected and manipulated at university labs, following the standard protocols established by each university (Universidade Federal do Rio de Janeiro, and Universidade do Estado do Rio de Janeiro), including the use of personal protective equipment.

### Flow cytometry assay

PBMCs were thawed in a water bath, and 1×10^6^ cells/tube were incubated with conjugated monoclonal antibodies: phycoerythrin (PE)-CD33; FITC-CD11b; PerCP-Cy5.5-HLA-DR; APC-Cy7-CD14; FITC-CD16; PE-CD56; and APC Cy7-CD3 (all from Biolegend, San Diego, California, USA). All samples were stained with a live/dead dye (BioRad, Hercules, California, USA) to exclude dead cells. Samples were acquired in FACS Canto II flow cytometer and analyzed using FlowJo software (version 10.4, TreeStar Inc., Ashland, USA). Supplementary Figure S1B-S1E shows a strategy of flow cytometry analyses. Briefly, singlet cells were first identified, and live PBMCs were selected. Subsequently, HLA-DR^low/−^ cells were gated to identify monocytic MDSCs as CD33^hi^CD11b^+^ and granulocytic MDSCs as CD33^low^CD11b^+^ (Supplementary Figure S1C). Live monocytes were defined to determine CD14^+^CD16^−^ classical monocytes, CD14^+^CD16^+^ intermediate monocytes, and CD14^int^CD16^+^ non-classical monocytes (Supplementary Figure S1D). Live lymphocytes were selected to identify CD3^+^ cells, followed by the identification of CD3^+^CD56^+^ NK T cells. Additionally, CD3^−^ cells were selected to delineate three NK cell populations: CD56^++^CD16^+−^, CD56^dim^CD16^+^, and CD56^-^CD16^+^ NK cells (Supplementary Figure S1E).

### Statistical analysis

Statistical analyses were performed using GraphPad-Prism software (version 8.0, GraphPad Software, Boston, USA). Results are expressed as median, and the significance was estimated using a nonparametric Mann-Whitney test. Spearman rank test was used to perform correlations among the variables. P<0.05 was considered significant.

## RESULTS

Table 1 shows the baseline characteristics of enrolled individuals. The median age for CALHIV and HU participants was 11 (6–17) and 13 (6–15) years, respectively. Among the CALHIV patients, 60% were on cART (median length of 1.2 years). At baseline, the viral load median was 1,811 (0–361,000) RNA copies/mL (the viral load remained detectable throughout the study in the same patients who were detected at baseline), CD4 count was 588 (378–1,432) cells/mm^3^, and nadir CD4 was 352 (0–708) cells/mm^3^. Participants were recruited in 2011, when the Brazilian guidelines recommended cART for children and adolescents belonging to clinical category B (moderate clinical signs and/or symptoms) or C (severe clinical signs and/or symptoms) of the Centers for Disease Control and Prevention (CDC), patients with CD4 T cell counting <350 cells/mm^3^ or <15%, or viral load >100,000 copies/mL^16^. Viral load and CD4 count assessments are not applicable to HU participants (Table 1).

**Table 1:** Demographic and baseline characteristics of children and adolescents living with HIV (CALHIV) and HIV-uninfected (HU) groups

Characteristic		CALHIV (N=10)	HU (N=6)
Sex			
	Female (%)	6 (60)	2 (33.3)
	Male (%)	4 (40)	4 (66.7)
Age (years)		11 (6–17)*	13 (6–15)*
On cART at entry (%)		60	NA
Length of cART (years, T0)		1.2 (0 –13.4)*	NA
Viral load (copies/mL, T0)		1,811 (0–361,000)*	NA
CD4 count (cells/mm^3^, T0)		588 (378–1,432)*	NA
% of CD4 (T0)		26,5 (17–35)*	NA
*Nadir* CD4 (cells/mm^3^, T0)		352 (0–708)*	NA

cART = combined antiretroviral therapy; NA = not applicable; T0 = before immunization (baseline).

* Median (range)

**Figure 1:**
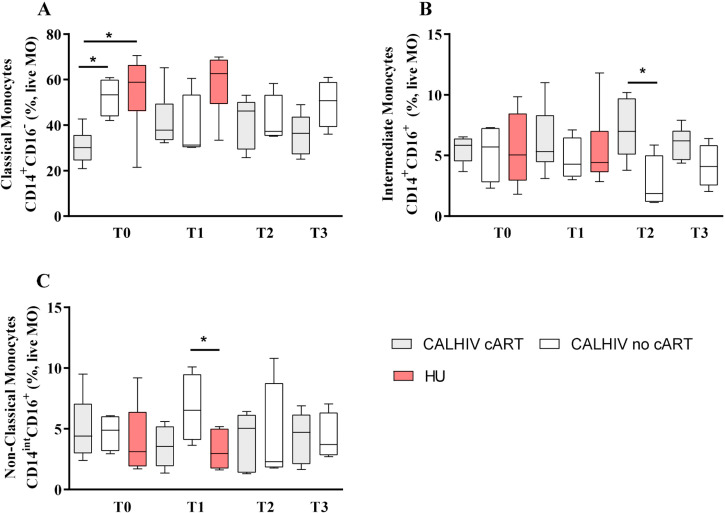
Altered frequency of monocytes in children and adolescents living with HIV. Frequency of classical monocytes (CD14^+^CD16^−^) (A), intermediate monocytes (CD14^+^CD16^+^) (B), and non-classical monocytes (CD14^int^CD16^+^) before immunization (T0), after the first MCC dose (T1), before the booster dose (T2), and after the second dose (T3). Monocyte populations are expressed as the frequency of live monocytes (MO). P-values were estimated employing the Mann-Whitney test. *p<0.05. cART = combined antiretroviral therapy, CALHIV = children and adolescents living with HIV, HU = HIV-uninfected.

The frequencies of myeloid and lymphoid cells were analyzed by flow cytometry (Supplementary Figure S1B-S1E). Generally, classical monocytes predominate among monocyte populations in both the CALHIV and HU groups at all time points evaluated (T0–T3). We found a lower frequency of classical monocytes in the CALHIV compared to the HU group across all time points assessed, especially at T1 (35.75% vs. 62.60%, respectively, p=0.033, Supplementary Figure S2A). On the other hand, a higher frequency of non-classical monocytes was observed among the CALHIV in all periods compared to the HU cohort, although statistical significance was not reached (Supplementary Figure S2C). We noted a lower frequency of classical monocytes in CALHIV on cART compared to those not receiving cART (30.15% vs. 53.30%, respectively, p=0.019) and compared to the HU group (30.15% vs. 58.90%, respectively, p=0.041) at baseline. Classical monocytes at T1 were less frequent in CALHIV not receiving cART compared to the HU group (31.30% vs. 62.60%, respectively, p=0.066) (Figure 1A). Alternatively, the frequency of non-classical monocytes in CALHIV not receiving cART was higher compared to the HU group at T1 (6.53% vs. 2.95%, respectively, p=0.038) (Figure 1C). Interestingly, a lower frequency of intermediate monocytes was found in CALHIV patients not on cART compared to those on cART at T2 (1.87% vs. 6.98%, respectively, p=0.038) (Figure 1B).

**Figure 2:**
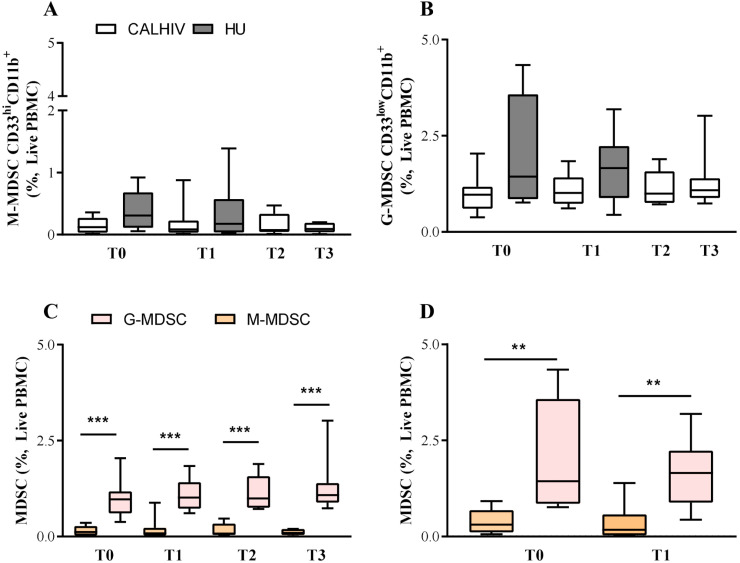
Frequency of myeloid-derived suppressor cells (MDSCs) in children and adolescents living with HIV (CALHIV) and in the HIV-uninfected (HU) group. Frequency of monocytic MDSC (M-MDSC - CD33^hi^CD11b^+^) (A) and granulocytic MDSC (G-MDSC - CD33^low^CD11b^+^) (B); Comparison of MDSC in CALHIV (C) and HU groups (D). MDSC populations are expressed as the frequency of live PBMC. P-values were estimated using Mann-Whitney test. **p<0.01. ***p<0.001. T0 = before immunization (baseline); T1 = 1–2 months after the first dose; T2 = about one year after T0; T3 = 1-2 months after the second dose.

Two distinct MDSCs subpopulations were observed in our cohort: granulocytic MDSCs (G-MDSCs – CD33^low^CD11b^+^) and monocytic MDSCs (M-MDSCs – CD33^hi^CD11b^+^)^17^. We did not find differences in the M-MDSCs or G-MDSCs across time between the CALHIV and HU groups (Figure 2A-2B). A higher frequency of G-MDSCs was observed in the CALHIV cohort (0.97%, 1.01%, 0.99%, and 1.08% for T0, T1, T2, and T3, respectively) compared to M-MDSCs (0.12%, 0.08%, 0.07%, and 0.09% for T0, T1, T2, and T3, respectively), p<0.001 (Figure 2C). The same profile was detected in the HU group (G-MDSCs 1.44% and 1.65% for T0 and T1, respectively, and M-MDSCs 0.31% and 0.17% for T0 and T1, respectively, p<0.01) (Figure 2D). A trend toward a lower frequency of M-MDSCs in the CALHIV group at T3 (0.09%) compared to the HU group at T0 (0.31%) was detected (p=0.053).

According to CD56 and CD16 expression, NK cells can be categorized into three populations, including the CD56^++^CD16^+−^, with elevated cytokine production and lower cytotoxic activity; the CD56^dim^CD16^+^, which exhibits high cytotoxic activity; and the CD56^−^CD16^+^, with lower cytokine production and cytotoxic activity^18^. Overall, a higher frequency of CD56^dim^CD16^+^ NK cells was observed across all periods evaluated in both CALHIV and HU groups (Supplementary Figure S3B). Notably, we found a higher frequency of CD56^−^CD16^+^ NK cells in CALHIV patients at T3 compared to the HU individuals at T1 (1.45% vs. 0.44%, respectively, p=0.049) (Supplementary Figure S3C). A trend toward a higher frequency of CD56^−^CD16^+^ NK cells in CALHIV patients compared to the HU group was noted at T1 (1.06% vs. 0.44%, respectively, p=0.056). In contrast, a lower frequency of CD56^dim^CD16^+^ was observed in CALHIV patients at T2 compared to the HU group at T0 (2.86% vs. 4.62%, respectively, p=0.056) (Supplementary Figure S3B). In the HU group, a tendency to a higher frequency of CD56^dim^CD16^+^ (4.62% vs. 2.18%) and total NK cells (6.25% vs. 3.25%) was observed at T0 compared to T1, respectively (p=0.065 for both) (Supplementary Figure S3B and S3D, respectively). A negative correlation between CD56^++^CD16^+−^NK cells and CD4 T cells frequency at baseline was observed (r= −0.64; p=0.050) in the CALHIV cohort. The same was observed between CD56^dim^CD16^+^ NK cells and CD4 T cells frequency (r= −0.63; p=0.053) (data not shown). No differences were observed in the frequency of CD56^++^CD16^+−^ NK or NKT cells during the period between the CALHIV and HU groups.

**Figure 3:**
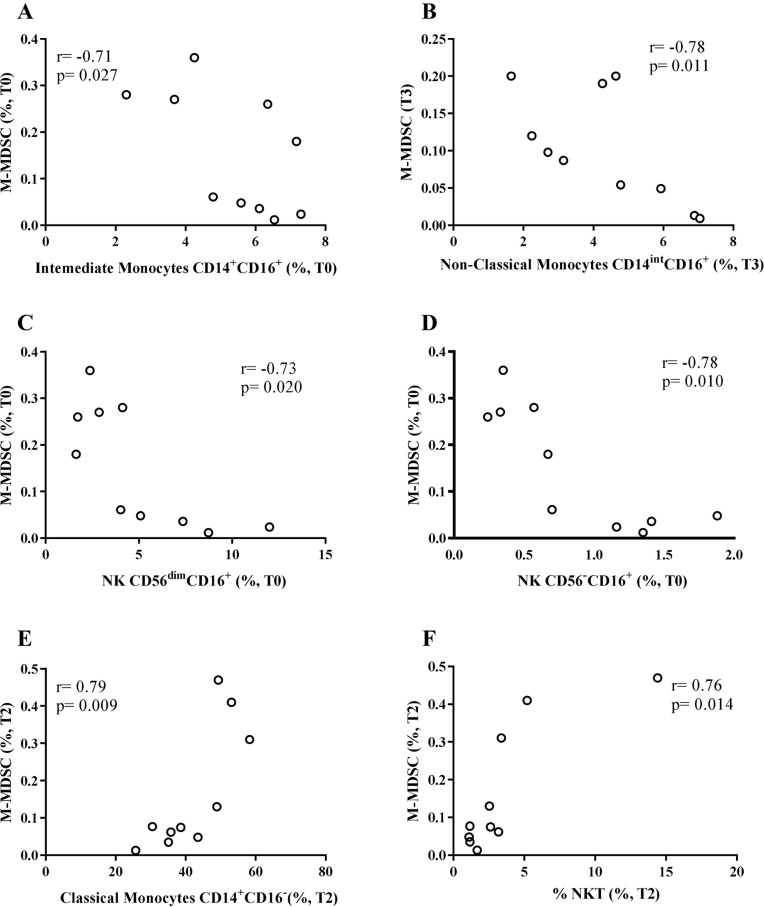
Correlation analysis of monocytic MDSCs (M-MDSCs) with innate-immune cells in CALHIV. M-MDSC inversely correlates with intermediate monocytes at baseline (T0) (A), with non-classical monocytes at T3 (B) and with NK CD56^dim^CD16^+^ (C) and NK CD56^-^CD16^+^ (D) at T0. At T2, M-MDSC positively correlates with classical monocytes (E) and NK-T cells (F). Correlations were evaluated using Spearman rank test. CALHIV = children and adolescents living with HIV; T0 = before immunization (baseline); T2 = about one year after T0; T3 = 1–2 months after the second dose.

**Figure 4:**
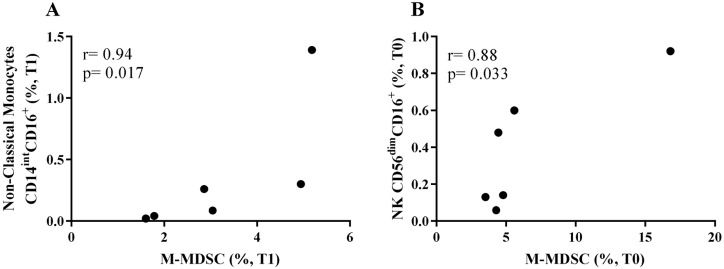
Correlation analysis of monocytic MDSCs (M-MDSCs) with innate-immune cells in the HU cohort. M-MDSC positively correlates with non-classical monocytes at T1 (A) and with NK CD56^dim^CD16^+^ at T0 (B). Correlations were evaluated using Spearman rank test. HU: HIV-uninfected. T0: before immunization (baseline); T1: 1–2 months after immunization.

Since MDSCs immunosuppresses via various mechanisms^8^, we performed correlations between G-MDSCs or M-MDSCs with the other innate-cell populations evaluated here. Concerning the CALHIV patients, we found a negative and significant correlation between M-MDSCs with intermediate monocytes (r= −0.71, p=0.027) at baseline (T0) (Figure 3A) and with non-classical monocytes (r= −0.78, p=0.011) at T3 (Figure 3B). M-MDSCs also negatively correlated with CD56^dim^CD16^+^ NK cells (r= −0.73, p=0.020) (Figure 3C) and with CD56^−^CD16^+^ NK cells (r= −0.78, p=0.010) (Figure 3D) at T0. A positive correlation was found between M-MDSCs and classical monocytes (r= 0.79, p=0.009) (Figure 3E) and NKT cells (r= 0.76, p=0.014) at T2 (Figure 3F). In contrast, for the HU group, we noted a positive correlation between non-classical monocytes with M-MDSCs at T1 (r= 0.94, p=0.017) (Figure 4A) and with CD56^dim^CD16^+^ NK cells at baseline (r= 0.88, p=0.033) (Figure 4B).

## DISCUSSION

HIV infection impairs both innate and adaptive immune responses, rendering children more susceptible to infections and less responsive to vaccines. Despite effective antiretroviral therapy, inflammation remains elevated, compromising immune function in treated patients^2^. In this study, we assessed the kinetics of monocytes, MDSCs, NK, and NKT cells in children and adolescents, both HIV-positive and HIV-negative, over approximately one year.

Alteration in monocyte subsets during HIV infection has been reported by different groups^19,20^. We detected a higher frequency of non-classical monocytes in the CALHIV group compared to the HU group in all periods evaluated, particularly in CALHIV patients not on cART, which could be driven by viral replication^20,21^. An increase in non-classical monocytes has been associated with non-AIDS-related diseases such as cardiovascular disease^22^. Our results also showed a lower frequency of classical monocytes in the CALHIV group compared to the HU cohort, as previously reported^19^. Classical monocytes are recruited to inflamed tissue, where they contribute to the immune response by recognizing microorganisms and producing proinflammatory cytokines^23^. Moreover, they serve as precursors to tissue macrophages and dendritic cells. A lower frequency of these cells may contribute to a generally poor immune response in HIV patients. The imbalance in monocyte population observed in this and other studies can be a reflection of the sustained immune activation observed in HIV patients despite cART^24^.

Natural killer (NK) cells are remarkable players during viral infections. HIV infection is linked to changes in NK cell subsets characterized by a reduction of CD56^+^ but an expansion of CD56^−^CD16^+^ NK cells^25–27^. In agreement with our results, Zulu and colleagues found no differences in the compartment of CD56^++^CD16^+−^ NK cells between CALHIV and HU groups. Although the differences were not found at all time points evaluated, we detected a higher frequency of CD56^−^CD16^+^ NK cells in CALHIV patients, corroborating the literature^25,26^. Moreover, the expansion of CD56^−^CD16^+^ NK cells was accompanied by a reduction in CD56^dim^CD16^+^ NK cells. CD56^−^CD16^+^ NK cells exhibit lower cytotoxic activity and cytokine production but possess inhibitory properties via the production of IL-10 and TGF-β, which may contribute to immunosuppression in HIV patients and increased susceptibility to infections^26,28^. Curiously, our results show an inverse correlation of CD56^dim^CD16^+^ and CD56^++^CD16^+−^ NK cells with CD4^+^ T cells frequency at baseline (T0). These results may reflect the heterogeneity of the CALHIV cohort, as most were under cART with suppressed viral load.

MDSCs suppress immune response via different mechanisms, such as nitric oxide, reactive oxygen species, and IL-10 production, among others^8^. Although we did not observe variation or differences between M-MDSCs and G-MDSCs over time in the CALHIV and HU groups, we found negative associations between M-MDSCs and certain cell types evaluated in this study. For example, in the CALHIV group, M-MDSCs were negatively associated with intermediate and non-classical monocytes, as well as with CD56^dim^CD16^+^ and CD56^−^CD16^+^ NK cells at distinct moments, which was not observed in the HU group. These negative correlations may reflect the overall disturbance caused by HIV infection.

## CONCLUSION

A limitation of our study is the heterogeneity among patients and the small sample size. Larger sample will be necessary in future studies. Another limitation was the lack of an evaluation of vaccine-induced serum bactericidal antibodies (SBA) production, as blood samples were collected prior to and 1–2 months after each MCC vaccine dose. Such investigation could have yielded valuable insights into the interplay between the cells analyzed and SBA production. Furthermore, it would be interesting to perform functional analyses of MDSCs to assess their suppressive activity.

The results presented here are preliminary but suggest a disturbance of innate immune compartments in children and adolescents living with HIV, which may contribute to their susceptibility to infection and poor vaccine response.
