# Supplementary material for: Innate-immune cell distribution in pediatric HIV patients and uninfected controls
Source: Rev Inst Med Trop Sao Paulo. 2024 Dec 16;66:e75. doi: 10.1590/S1678-9946202466075 (PMC11654119; doi:10.1590/S1678-9946202466075)
Supplement: Supplementary file 1 [file 1678-9946-rimtsp-66-S1678-9946202466075suppl01.pdf]

REVISTA DO INSTITUTO DE MEDICINA TROPICAL DE SÃO PAULO

JOURNAL OF THE SÃO PAULO INSTITUTE OF TROPICAL MEDICINE

SUPPLEMENTARY MATERIAL

https://doi.org/10.48331/scielodata.AYLFE3

Innate-immune cell distribution in pediatric HIV patients and uninfected controls

Cynthia Oliveira Aquino[1*], Fernanda Mariz Pereira[1*], Ana Cristina Cisne Frota[2], Cristina Barroso Hofer[2,3], Lucimar Gonçalves Milagres[1], Wânia Ferraz Pereira Manfro

[1]Universidade do Estado do Rio de Janeiro, Departamento de Microbiologia, Imunologia e Parasitologia, Rio de Janeiro, Rio de Janeiro, Brazil

[2]Universidade Federal do Rio de Janeiro, Instituto de Puericultura e Pediatria Martagão Gesteira, Rio de Janeiro, Rio de Janeiro, Brazil

[3]Universidade Federal do Rio de Janeiro, Departamento de Medicina Preventiva, Rio de Janeiro, Rio de Janeiro, Brazil

*These authors contributed equally to the article

Correspondence to: Wânia Ferraz Pereira Manfro
Universidade do Estado do Rio de Janeiro, Departamento de Microbiologia, Imunologia e Parasitologia, Av Prof. Manoel de Abreu, 444, 3º andar, CEP 20550–170, Rio de Janeiro, RJ, Brazil
Tel: +55 21 2868-8280

E-mail: waniafpm@gmail.com

Received: 25 June 2024

Accepted: 7 November 2024

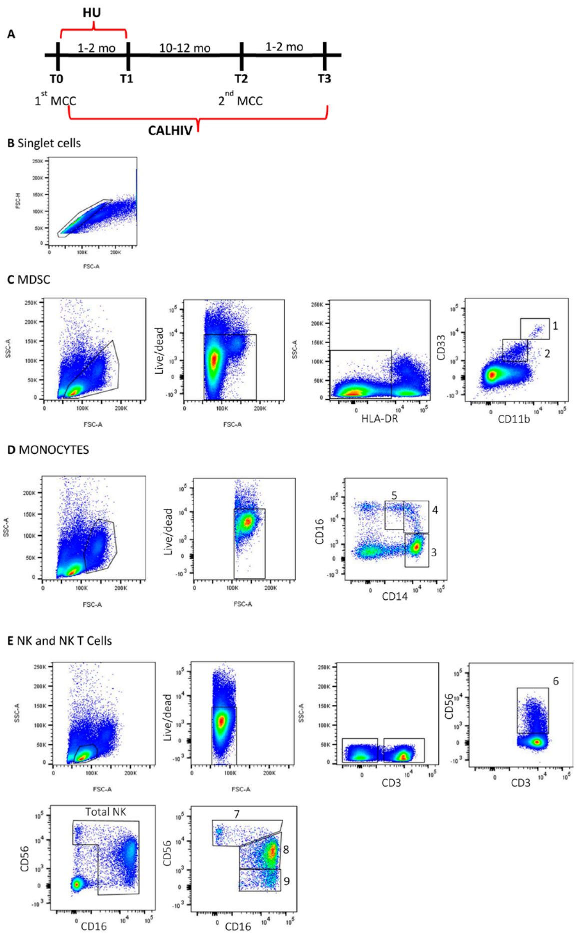

Supplementary Figure S1 - Blood sample collection, immunization, and strategy of flow cytometry analysis from one representative experiment with PBMCs samples of a control individual: (A) Blood samples were collected before immunization (T0, baseline) when the first dose of MCC was administered, and 1–2 months after the first dose (T1). A second dose of MCC (T2) was administered about one year after T0 only to CALHIV patients, and the blood sample was collected at T2 and 1–2 months after the second dose (T3); (B) Singlet cells were selected to exclude the doublet cells; (C) PBMC were gated and live PBMC were selected to identify HLA-DR$^{low/-}$, then characterize monocytic MDSC as CD33^{hi}CD11b^{+} (1) and granulocytic MDSC as CD33^{low}CD11b^{+} (2); (D) The region of monocytes were gated, and live monocytes region were defined to determine CD14^{+}CD16^{-} classical monocytes (3), CD14^{+}CD16^{+} intermediate monocytes (4), and CD14^{int}CD16^{+} non-classical monocytes (5); (E) The region of lymphocytes were determined and live lymphocytes were selected to identify CD3^{+} cells, then characterize CD3^{+}CD56^{+} NK T cells (6). CD3^{-} cells were selected to identify three populations of NK cells: CD56^{++}CD16^{+-} (7), CD56^{dim}CD16^{+} (8), and CD56^{-}CD16^{+} (9) NK cells. Total NK cells are also shown. Analyses of HIV-infected individuals followed the same pattern. MCC: meningococcal C conjugate vaccine; HU: HIV-uninfected; CALHIV: children and adolescents living with HIV; PBMCs: peripheral blood mononuclear cells.

This is an open-access article distributed under the terms of the Creative Commons Attribution License.

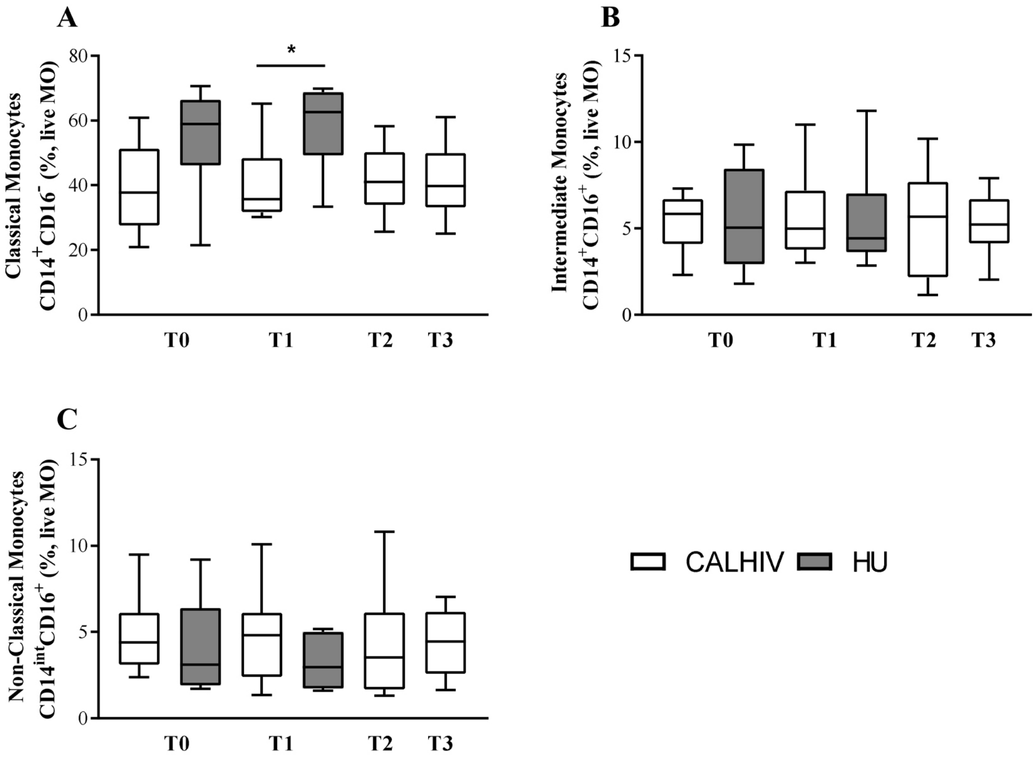

Supplementary Figure S2 - Frequency of monocytes in children and adolescents living with HIV (CALHIV) and in the HIV-uninfected (HU) group. Frequencies of classical monocytes ($CD14^{+}CD16^{-}$) (A), intermediate monocytes ($CD14^{+}CD16^{+}$) (B), and non-classical monocytes ($CD14^{int}CD16^{+}$) (C) were determined by flow cytometry. Blood samples were collected before immunization (T0), 1–2 months after the first MCC dose (T1), about one year after T0 when a booster dose was administered only to HIV infected patients (T2), 1–2 months after the second dose of MCC (T3). Monocyte populations are expressed as the frequency of live monocytes (MO). P-values were estimated using Mann-Whitney test. *$p<0.05$. MCC = meningococcal C conjugate vaccine.

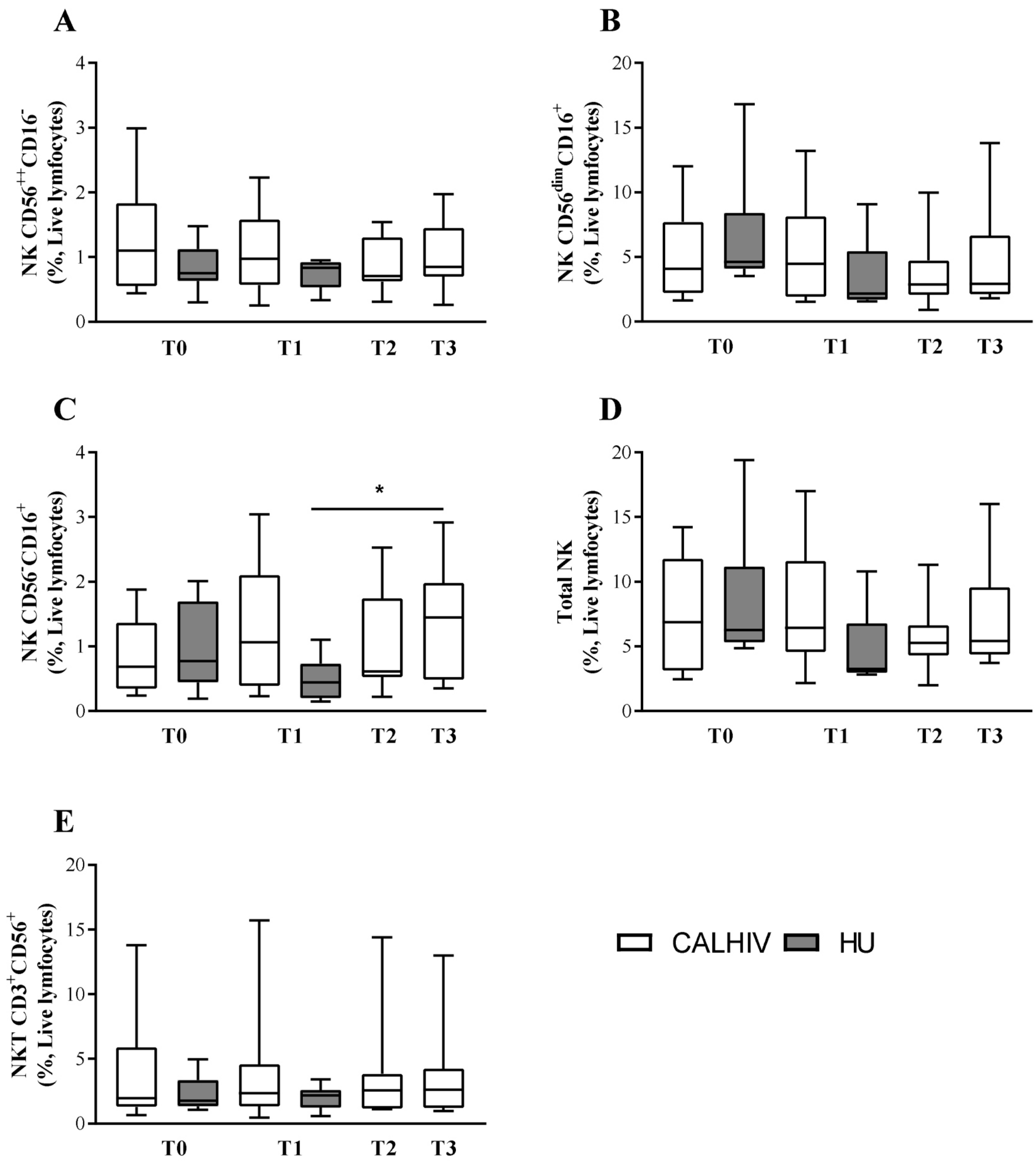

Supplementary Figure S3 - Frequency of natural killer cells. A: NK cells $CD56^{++}CD16^{+-}$, B: NK cells $CD56^{dim}CD16^{+}$, C: NK cells $CD56^{-}CD16^{+}$, D: total NK cells, E: NK-T cells $CD3^{+}CD56^{+}$. Natural killer cells are expressed as the frequency of live lymphocytes. CALHIV = children and adolescents living with HIV; HU = HIV uninfected; NK = natural killer cells; NKT = natural killer T-cells. T0 = before immunization (baseline); T1 = 1–2 months after the first dose; T2 = about one year after T0; T3 = 1–2 months after the second dose. P-values were estimated using Mann-Whitney test. *$p<0.05$.
